# The evolving role of extracellular vesicles (exosomes) as biomarkers in traumatic brain injury: Clinical perspectives and therapeutic implications

**DOI:** 10.3389/fnagi.2022.933434

**Published:** 2022-10-06

**Authors:** Naushad Ahmad Khan, Mohammad Asim, Ayman El-Menyar, Kabir H. Biswas, Sandro Rizoli, Hassan Al-Thani

**Affiliations:** ^1^Clinical Research, Trauma Surgery Section, Department of Surgery, Hamad General Hospital, Doha, Qatar; ^2^Department of Clinical Medicine, Weill Cornell Medical College, Doha, Qatar; ^3^Division of Biological and Biomedical Sciences, College of Health and Life Sciences, Hamad Bin Khalifa University, Qatar Foundation, Doha, Qatar; ^4^Trauma Surgery Section, Department of Surgery, Hamad General Hospital, Doha, Qatar

**Keywords:** extracellular vesicles, exosomes, traumatic brain injury, neurodegenerative diseases, blood–brain barrier, biomarkers

## Abstract

Developing effective disease-modifying therapies for neurodegenerative diseases (NDs) requires reliable diagnostic, disease activity, and progression indicators. While desirable, identifying biomarkers for NDs can be difficult because of the complex cytoarchitecture of the brain and the distinct cell subsets seen in different parts of the central nervous system (CNS). Extracellular vesicles (EVs) are heterogeneous, cell-derived, membrane-bound vesicles involved in the intercellular communication and transport of cell-specific cargos, such as proteins, Ribonucleic acid (RNA), and lipids. The types of EVs include exosomes, microvesicles, and apoptotic bodies based on their size and origin of biogenesis. A growing body of evidence suggests that intercellular communication mediated through EVs is responsible for disseminating important proteins implicated in the progression of traumatic brain injury (TBI) and other NDs. Some studies showed that TBI is a risk factor for different NDs. In terms of therapeutic potential, EVs outperform the alternative synthetic drug delivery methods because they can transverse the blood–brain barrier (BBB) without inducing immunogenicity, impacting neuroinflammation, immunological responses, and prolonged bio-distribution. Furthermore, EV production varies across different cell types and represents intracellular processes. Moreover, proteomic markers, which can represent a variety of pathological processes, such as cellular damage or neuroinflammation, have been frequently studied in neurotrauma research. However, proteomic blood-based biomarkers have short half-lives as they are easily susceptible to degradation. EV-based biomarkers for TBI may represent the complex genetic and neurometabolic abnormalities that occur post-TBI. These biomarkers are not caught by proteomics, less susceptible to degradation and hence more reflective of these modifications (cellular damage and neuroinflammation). In the current narrative and comprehensive review, we sought to discuss the contemporary knowledge and better understanding the EV-based research in TBI, and thus its applications in modern medicine. These applications include the utilization of circulating EVs as biomarkers for diagnosis, developments of EV-based therapies, and managing their associated challenges and opportunities.

## Introduction

Neurodegenerative diseases (NDs) are debilitating diseases that affect the nervous system and are closely related to brain function (Hansson, 2021). These diseases mainly include acute injury to the central nervous system (CNS), such as traumatic brain injury (TBI), stroke, and chronic diseases (Alzheimer's and Parkinson's disease; Meisel et al., 2005; Rehman et al., 2019; Guedes et al., 2020a). Currently, the diagnosis of NDs is primarily reliant on neuroimaging techniques, such as computed tomography (CT) and magnetic resonance imaging (MRI), which involve high cost and biochemical examination of cerebrospinal fluid (CSF) (Mattsson et al., 2017; Anderson et al., 2019; Jia et al., 2019). Furthermore, NDs lack specific symptoms and usually have a long course and a slow onset, making their early diagnosis and treatment a challenging task (Niu et al., 2020). The current blood-based biomarkers have some limitations, such as their inefficiency in detecting or identifying alternations in the preliminary or early phases of CNS diseases (Jarmalavičiute and Pivoriunas, 2016; Chen J. J et al., 2017; Xia et al., 2019). In addition, there are few or not recognized effective therapeutic treatments for reversing or treating this category of disorders (Paranjpe et al., 2019). Therefore, there is a pressing need to explore reliable biomarkers for diagnosis and therapy of the acute and chronic stages of neurodegenerative diseases.

Cellular crosstalk underpins many clinical conditions and neurological disorders within the CNS. Even though several mechanisms have been discovered as initiators of the pathogenesis of the disease, it is now evident that uncontrolled neuroinflammation and consequent cellular injury are the major defining aspects of numerous neurological diseases. Extracellular vehicles (EVs), a heterogeneous family of membrane-bound vesicles (size < 1 μm), are produced through the cell's paracrine pathway and are the newest addition to the intracellular communication system (Budnik et al., 2016; Pegtel and Gould, 2019; Wu et al., 2021). EVs are secreted by different cells (i.e., lymphocytes, platelets, astrocytes, fibroblasts, endothelium, and neurons). They may be found in all biological fluids, including blood, urine, saliva, breast milk, and CSF (Fauré et al., 2006; Kumari and Anji, 2022). Moreover, EVs are relatively stable in a variety of physiological conditions and can preserve biomolecules (proteins and miRNA) in the extracellular environment from breakdown and denaturation (Mulcahy et al., 2014; Ha et al., 2016; Kalluri and LeBleu, 2020; Di Bella, 2022). Emerging data suggest that they can serve as biomarkers of NDs that are more consistent than CSF, blood, or urine (Andjus et al., 2020; Pinnell et al., 2021).

EVs have evolved as multifaceted signaling molecules that may alter the phenotypic traits of target cells in multiple ways, such as the initiation of signaling events at the cell surface and direct transfer of bio-compatible and active material between cells (Colombo et al., 2014). EVs also include a variety of biological payloads, such as membrane, cytosolic proteins, micro RNA (miRNA), long non-coding RNA (lncRNA), and sometimes even mitochondrial DNA (mtDNA) (Colombo et al., 2014; Takahashi et al., 2017). They act as a selective transporter of proteins, lipids, and genetic materials and form a significant intracellular communication system capable of regulating a variety of cellular functions in recipient cells. Furthermore, EVs can also alter the biochemical composition of the extracellular environment (Iraci et al., 2017) and maintain the cellular homeostasis of the cells secreting EVs (Takahashi et al., 2017).

EVs produced by neuronal and glial cells are integral to the intricate system of interrelated signals underpinning the physiology and pathophysiology of the central nervous system (Krämer-Albers and Hill, 2016). Emerging data show that EV-regulated intracellular signaling may help modulate neuronal activity and myelin formation (Antonucci et al., 2012). EVs may contribute to the propagation of toxic misfolded proteins in NDs and influence the aggregation process and aggregate clearance (Thompson et al., 2016; Holm et al., 2018). It has been demonstrated that EVs may cross the blood–brain barrier (BBB) in injured patients, indicating the spread of neuroinflammation with systemic repercussions (Kumar et al., 2017; Saint-Pol et al., 2020; Shao et al., 2022). The capacity of EVs to traverse the BBB, along with their low immunogenicity, provides a practical basis for their use as excellent biomarkers and ideal candidates for drug delivery carriers for the therapy and management of CNS diseases.

This review summarizes the current biology and knowledge of EV research and discusses the roles and shreds of evidence of EVs in the pathogenesis of NDs, with special emphasis on TBI. Also, we discuss the application of EVs in modern medicine, such as the utilization of circulating EVs as biomarkers for diagnosis, emphasizing novel biomarkers in NDs illness, and developments of EV-based therapies. The majority of research conducted so far has mainly concentrated on microvesicles and exosomes. The mounting attention to EVs has resulted in the development of specific EV databases, such as Exocarta and miRandola, that assemble data on EVs composition and are continually updated with published literature in this field (Greening et al., 2017; Kalluri and LeBleu, 2020). The current narrative review emphasizes EVs with a special focus on their biological significance as biomarkers of TBI.

## The nanoscale in brief: Subtypes, biochemical features, biogenic origin, and molecular cargos

Despite significant advances in the biology of EVs research over the last two decades, the specific mechanisms responsible for regulating their biogenesis, cargo loading, transfer, and release in the extracellular environment to facilitate cell-to-cell communication remain incompletely defined and understood. The following properties are shared by all varieties of EVs: a lipid bilayer membrane enclosing a fluid-filled vesicle, intraluminal transfer of cargo containing proteins, nucleic acid, lipids, and their subsequent release into the extracellular environment (Lötvall et al., 2014; Théry et al., 2018; Russell et al., 2019). In the literature, EVs subtypes have been divided into microvesicles or microparticles having a diameter of up to 1,000 nm and exosomes with a diameter of <100 nm. Although the contemporary literature has focused on the size range and function-based characterization of EVs, the recent consensus has acknowledged that exploring the biogenesis and variability in the molecular compositions of different EV subtypes may also be important (Lötvall et al., 2014; Théry et al., 2018; Gurunathan et al., 2019; Russell et al., 2019). Characteristics of different subtypes of EVs are listed in Table 1.

**Table 1:** Characteristics of extracellular vehicles (EVs) subtypes.

**EVs subtype**	**Biogenesis**	**Morphology**	**Markers**	**Cellular/molecular cargos**	**References**
**Exosomes** Size range: 40–150 nm diameter	The membrane of early endosomes bulges out to make MVBs in endosomes. Exosomes are made when MVBs fuse with the plasma membrane and is the subsequent release of intraluminal vesicles in the extracellular domain	Normally appear cup-shaped on transmission-electron microscopy	• Endosome-associated components: Annexins, flotillins, integrins • ESCRT, CD9, CD81, CD82, and TSG101	• Surface proteins, cell-specific markers adhesion molecules, MHC class I and II • Neurotransmitter • Receptors and Lipids. • Nucleotides: coding RNA (mRNA), non-coding RNA (miRNAs, circRNAs, lncRNAs)	Greening et al., 2017; Kalluri and LeBleu, 2020
**Microvesicles** Size range: 100–1,000 nm diameter	Outward budding of plasma membrane	Spherical, discoid, and cylindrical	• Considerable overlap with Exosomes. • No definite markers. • Selectins, integrins	• Surface proteins, intraluminal proteins • adhesion proteins • integrins, Selectins. • Nucleotides: coding RNA (mRNA), non-coding RNA (miRNAs, circRNAs, lncRNAs). • Lipids (Phosphatidyl-serine, sphingomyelin)	Colombo et al., 2014; Janas et al., 2016
**Apoptotic bodies** Size range: 500–5,000 nm diameter	Shedding/budding of the plasma membrane by cells experiencing apoptosis	Heterogeneous	• Phosphatidylserine	• DNA, histones, and cytoplasmic components, adhesion proteins	Gurunathan et al., 2019; Kang et al., 2021; Obeng, 2021

### Exosomes

Exosomes are membrane-derived, globular, intraluminal, nanoscale vesicles of about size range of 40–150 nm in diameter released by several types of cells (e.g., neurons, adipocytes, endothelial and epithelial cells, astrocytes, B-lymphocytes, mast cells, and dendritic cells) during normal cellular activity and, more specifically, in response to cellular stress factors (Greening et al., 2017; Van Niel et al., 2018; Kalluri and LeBleu, 2020). Exosomes have been shown to act as transport vehicles for nucleic acids, such as coding and non-coding RNA forms, along with functional proteins and cellular metabolites (Takahashi et al., 2017; Jeppesen et al., 2019; Kalluri and LeBleu, 2020). Exosomes have been isolated in several biological fluids, including blood, CSF, urine, semen, breast milk, amniotic fluid, bronchial fluid, and lymph in healthy and pathological conditions (Greening et al., 2017; Takahashi et al., 2017; Kalluri and LeBleu, 2020). Initially, it was considered as non-functional tiny vesicles just eliminating redundant proteins and other metabolites from the cells and discharging them. Hence, they were initially thought to contain “cellular junk” and function merely as garbage transport and disposals for undesirable proteins and molecules (Harding et al., 1983). Later, it was discovered that they have an immunological function, in which they act as a mode of intracellular communication, and play an important role in normal physiological processes, such as immunogenicity, inflammation, and nerve function (Chivet et al., 2013; De Rivero Vaccari et al., 2016; Anakor et al., 2021).

Exosomes are considered intraluminal vesicles formed by the inward budding of membrane-bound endosomes and their subsequent discharge into the multivesicular body (MVB). Exosomes are released from cells when MVBs merge with either the lysosome, which degrades their contents, or the plasma membrane and releases their contents (Colombo et al., 2014). The MVBs are transferred to the plasma membrane, followed by fusion and release of contents into the extracellular domain (Heijnen et al., 1999). Transmembrane proteins are integrated into the invaginating membrane during this process, preserving a structural orientation identical to the plasma membrane (Gurung et al., 2021). A heteromeric protein complex known as endosomal sorting complex is required for transport (ESCRT) along with related proteins [e.g., programmed cell death 6 interacting protein; (also known as ALIX), tumor susceptibility gene 101 protein; TSG101] and sphingolipids or tetraspanins that are involved in the tightly controlled mechanisms for cargo selection, inward budding process, and intraluminal vesicle production (van Niel et al., 2011; Kowal et al., 2016; Willms et al., 2016). Additionally, the ESCRT-independent pathway works in concert with ESCRT-dependent mechanisms to produce exosomes (Doyle and Wang, 2019). Exosomes have been identified to contain endosome-linked components such as Annexins and flotillins, as well as ESCRT and associated proteins such as TSG101 and ALIX (Théry et al., 2018; Jadli et al., 2020; Gurung et al., 2021; Wei et al., 2021). Exosomes are also rich in membrane proteins that play critical functions in forming endosomes or MVBs, such as tetraspanins CD9, CD81, and CD63, which are considered exosome-specific markers involved in exosome biogenesis (Xu et al., 2016; Théry et al., 2018; Jadli et al., 2020).

## Microvesicles or microparticles

MVs, also referred to as ectosomes or microparticles, are subtypes of EVs that are larger than exosomes, typically ranging between 100 and 1,000 nm in diameter and containing cytoplasmic material (Colombo et al., 2014; Janas et al., 2016). Like exosomes, MVs can originate from a variety of cell types, with their lipid and protein composition indicating their biological origin. In addition to their smaller size, microvesicles are distinct from exosomes in their underlying biogenesis mechanism. They are directly formed by the plasma membrane's outward blebbing and discharge the nascent MV into the extracellular environment (Tricarico et al., 2017). However, the route of biogenesis and cargo loading process of MVs are not as well defined and understood as exosomes. They also contain varying amounts of adhesion molecules, such as integrins, which influences vesicle transport and uptake. It has been demonstrated that MVs transport molecular cargoes consisting of many functional proteins, nucleic acids, and bioactive lipid molecules to the cell of their origin. When discharged into the extracellular space, MVs enter the circulation, carrying their molecular payloads/cargo to neighboring or far away cells and initiating phenotypic and functional changes relevant to various NDs. Due to their overlapping physiochemical features, lack of differentiating markers, and comparable biological contents makes it difficult to separate exosomes and microvesicles experimentally (Pathan et al., 2019). Even though tetraspanins are considered unique markers for exosomes, these proteins have been identified in microvesicles and other vesicles recently (Crescitelli et al., 2013; Tauro et al., 2013). A study by Jeppesen et al. (2019) reported annexin A1 as a microvesicle specific biomarker. More experimental data containing robust characterization techniques are needed to identify specific proteins enriched in MVs and distinguish them from other EV subtypes.

## Apoptotic bodies

Apoptotic bodies include a heterogenous group of cell particles produced by dying cells in the extracellular space and are a subset of EVs released by apoptotic cell plasma membranes formed during apoptosis (Hill, 2019). They are relatively large EVs with diameters ranging from 500 to 5000 nm, fragmented subcellular organelles for degradation, and variable morphology. These vesicles include subcellular organelle breakdown products, such as DNA, histones, and cytoplasmic components (Gurunathan et al., 2019; Kang et al., 2021; Obeng, 2021). Because apoptotic bodies are digested by phagocytic cells, they do not participate in intercellular communication, such as exosomes and microvesicles.

A proper understanding of different subtypes of EVs is crucial in studying physiological functions. It is technically challenging and tricky to characterize the subpopulations of EVs (Zabeo et al., 2017; Tkach et al., 2018). Considering the methodological constraints to accurately characterize EVs, we shall use the term EVs in the subsequent portions of this review except in studies where their biogenic origin has been unambiguously confirmed.

## EV cargos: Nucleic acids, proteins, and lipids

The field of EV research is continuously evolving, and efforts are ongoing to quantify the different proportions of genetic material (nucleic acids) found in EVs. However, the accurate classification seems to be contingent on the methodological approach involved in their isolation and, in particular, the specific cell type being analyzed. EVs carry multiple types of genetic material, particularly all subtypes of RNA, which include mRNA, transfer RNA, miRNA, lncRNA, small nuclear, ribosomal, and cytoplasmic RNA (Bellingham et al., 2012a; Yáñez-Mó et al., 2015; Wortzel et al., 2019; Amiri et al., 2022). Some mRNAs are more abundant in EVs than cells, and some data suggest disease-specific patterns of vesicular miRNA (Huang et al., 2013). In addition to various species of RNA, a fraction of EVs, particularly tumor-derived EVs, have been found to contain DNA (both single and double-stranded) transposons, nuclear, and mitochondrial DNA (mtDNA) (Guescini et al., 2010; Li et al., 2021). Microvesicles produced by glioblastoma cells include mRNA, miRNA, and proteins. Normal host cells, such as brain microvascular endothelial cells, take up these microvesicles. It has been demonstrated that they can carry RNA and proteins that stimulate tumor development (Skog et al., 2008). These microvesicles are also rich in angiogenic proteins, which drive tubule development in endothelial cells. Thus, they may also function as a vehicle for transmitting genetic information and proteins to recipient cells in the tumor environment (Valadi et al., 2007).

MiRNAs, one of the most significant EVs cargo, are non-coding RNAs (consisting of 15–20 nucleotides) and are important players in controlling protein expression and have been demonstrated to be highly concentrated in exosomes, indicating a selective method of miRNA loading during vesicle synthesis (Guduric-Fuchs et al., 2012; Yáñez-Mó et al., 2015; Beatriz et al., 2021). However, the precise underlying mechanism of packaging, enrichment, and release into the recipient cell is not well known, and the utility of EVs-derived miRNAs as consistent indicators of disease progression is still under investigation.

The protein composition of EVs varies from cell to cell and is heavily influenced by their biological origin. EVs include around 40,000 proteins, accounting for roughly one-quarter of the known human proteome (Keerthikumar et al., 2016; Duong et al., 2019). Endosomal membrane–localized proteins involved in biogenesis are the most prevalent in EVs. The ESCRT heteromeric protein complex components, including TSG101, ALIX, flotillins, and tetraspanins, are among the proteins in EVs (CD81, CD82, CD63, and CD9) (Guedes et al., 2020a; Gurung et al., 2021). Most of the above-mentioned membrane-associated proteins used as markers of different subpopulations of EVs (e.g., exosomes) are not limited to neuronal cells. However, they may also be detected in other EVs (e.g., MVs) (Lötvall et al., 2014).

Apart from protein and nucleic acids molecules, EVs also have lipids molecules (e.g., cholesterol, phospholipids, glycerophospholipids, and sphingolipids) and some biologically active lipid molecules (e.g., leukotrienes, prostaglandins, and phospholipase C), which act as lipid carriers facilitating the transfer of biologically active lipid molecules to a target cell (Skotland et al., 2017). EVs communicate with target cells *via* interaction between receptors and ligands at the surface of the membrane, through endocytosis, or fusing directly with the plasma membrane (Rojas et al., 2020). The structure of distinct subtypes of EVs is recognized to transport a range of important nucleic acids, functional proteins, and lipids, which may be transferred to target cells and translated into the corresponding proteins, resulting in epigenetic alterations. These functions of molecular cargo of EVs have led to the innovative notion that they can act as mediators of intercellular communication, which has been utilized and expanded in various disciplines, including the bench to the clinic (Coleman and Hill, 2015; Hsu et al., 2022; Weng et al., 2022).

## Role of EVs in the maintenance and repair of the CNS

A substantial body of evidence for the EVs involvement in the maintenance of CNS physiological functions comes from studies concentrating on brain/neuronal cells. Neuronal cells produce and release EVs, and many glial cell types regulate or assist neuronal processes. For example, in response to the neurotransmitter glutamate, oligodendrocytes, a glial cell engaged in myelination of the axon, produce EVs in a calcium-dependent (Ca^2^+) way (Frühbeis et al., 2013a). Neuronal activity, in turn, leads to an increase in oligodendrocyte EV secretion, which protects neurons from oxidative damage and malnutrition by enhancing their metabolic activity (Frühbeis et al., 2013a). However, it should be recognized that EV oligodendrocytes can interact with cell types other than neurons. For instance, a subset of microglial cells may internalize and destroy oligodendrocyte EVs (Fitzner et al., 2011), implying the quantity of oligodendrocyte-derived EVs available to control neuronal actions may be dependent on microglial function. Consequently, neuronal EVs may transmit bioactive molecular payloads such as proteins, nucleic acids, and lipids across cells, modulating neurovascular integrity, preserving synaptic function, and maintaining myelination.

Microglia, a subtype of glial cell, acts as the initial line of defense following brain injury and releases exosomes enriched within aminopeptidase CD13, which are metabolically active in catabolismleucine- and methionine-enkephalins peptides (a neuropeptide) (Potolicchio et al., 2005). A common element in EV-mediated neuron–glia communication is that EV release seems to be linked with synaptic activity (Frühbeis et al., 2013a; Chivet et al., 2014; Fröhlich et al., 2014; Laulagnier et al., 2017). Emerging data suggest that the relationship between neuronal EVs discharge and synaptic activity may be important for the maintenance of plasticity, implying that neuronal EVs might influence synaptic plasticity and play an important role in maintaining neurovascular integrity (Korkut et al., 2013; Krämer-Albers and Hill, 2016; Blanchette et al., 2022). The trafficking of certain RNAs into EVs appears to have a function, such as the maintenance of synaptic plasticity and its association with local protein synthesis (Goldie et al., 2014; Anakor et al., 2021). Neuronal-derived EVs can also transport their molecular cargo to other brain cells, influencing their behavior and potentially affecting the synaptic activity and neurovascular integrity. The production of EVs from activated neurons may assist in removing less functional synapses and neuronal remodeling (Xu et al., 2017), suggesting that the transfer of EVs mediated regulatory components may have a crucial role in safeguarding the functional integrity of the CNS.

Glial cells derived EVs have been found to control neuronal activity and offer neurons support and assistance on synaptic action *via* activating sphingolipid metabolism. When glutamate is present, oligodendrocytes release EVs, which include MVs at the axonal surface (Frühbeis et al., 2013a). Glial EVs may potentially aid neurons' energy metabolism by delivering enzymes and substrates of the glycolytic pathway to neurons during synaptic action (Budnik et al., 2016). Furthermore, some proteomic studies have found that EVs derived microglia and oligodendrocytes are involved in the transport of numerous enzymes of energy metabolism (Potolicchio et al., 2005; Drago et al., 2017). EVs are also involved in myelination remyelination. EVs from multiple origins were found to perform an important role in the myelination process (Domingues et al., 2020), indicating that EVs from a peripheral origin might participate in and regulate the process of myelination and its maintenance under specific circumstances. Schwann cells, which are part of the peripheral nervous system and release exosomes, have been shown to promote the maintenance and regeneration of local axonal cells (Lopez-Leal and Court, 2016). Moreover, it has been shown that the miRNA from Schwann cells in axon terminals is mediated through exosomes, impacting gene expression and neurite growth (Ching et al., 2018).

## Role of the blood–brain barrier in the transportation of EVs to and from CNS

The BBB is a unique microvasculature of the CNS, which is a very selectively permeable interface of capillary endothelial cells that connects the circulatory system to the brain's extracellular environment and facilitates the communication between the periphery and CNS (Naranjo et al., 2021). BBB closely regulates CNS homeostasis, which is necessary for appropriate neuronal function as well as for protecting the CNS from infections, toxins, inflammation, and injury (Obermeier et al., 2016). It is now widely acknowledged that EVs can breach the complex BBB and play a central role to initiate, promote, and reinforce physiological blood-to-brain transport in several pathological chronic processes such as NDs including TBI (Pegtel et al., 2014; Saint-Pol et al., 2020; Busatto et al., 2021). Therefore, understanding the processes by which EVs interact with the BBB under normal and pathological settings might lead to the creation of novel vehicles for targeted brain delivery, as well as the identification and validation of diagnostic and prognostic biomarkers of brain diseases. Due to the BBB's impediment to medication delivery to the CNS, significant attempts have been undertaken to develop techniques to modify or bypass the BBB to deliver medicines (Rufino-Ramos et al., 2017; Shaimardanova et al., 2020).

When discussing the neuronally generated vesicles, especially in the periphery, we have to explore whether EVs cross the BBB in either direction. Many studies have shown that EVs are transported over the BBB and reach the periphery; however, the underlying mechanism remains unclear, and evidence is sparse (Pegtel et al., 2014; Yáñez-Mó et al., 2015; Saint-Pol et al., 2020). Recent studies have shown that anti-inflammatory drugs could be delivered to the mice's brains through intranasal injection of EVs, indicating that EV administration to the CNS is feasible (Zhuang et al., 2011; Loch-Neckel et al., 2022). Similarly, the systemic administration has also exploited EVs to transfer small-interfering RNA molecules to mice brains (Cooper et al., 2014). Because BBB failure is a recognized characteristic of AD (Cooper et al., 2014) and TBI (Guedes et al., 2020a; Zhai et al., 2021), EV transfer from peripheral/systemic circulation to the brain may have therapeutic promise in NDs (Yamazaki and Kanekiyo, 2017).

**Figure 1:**
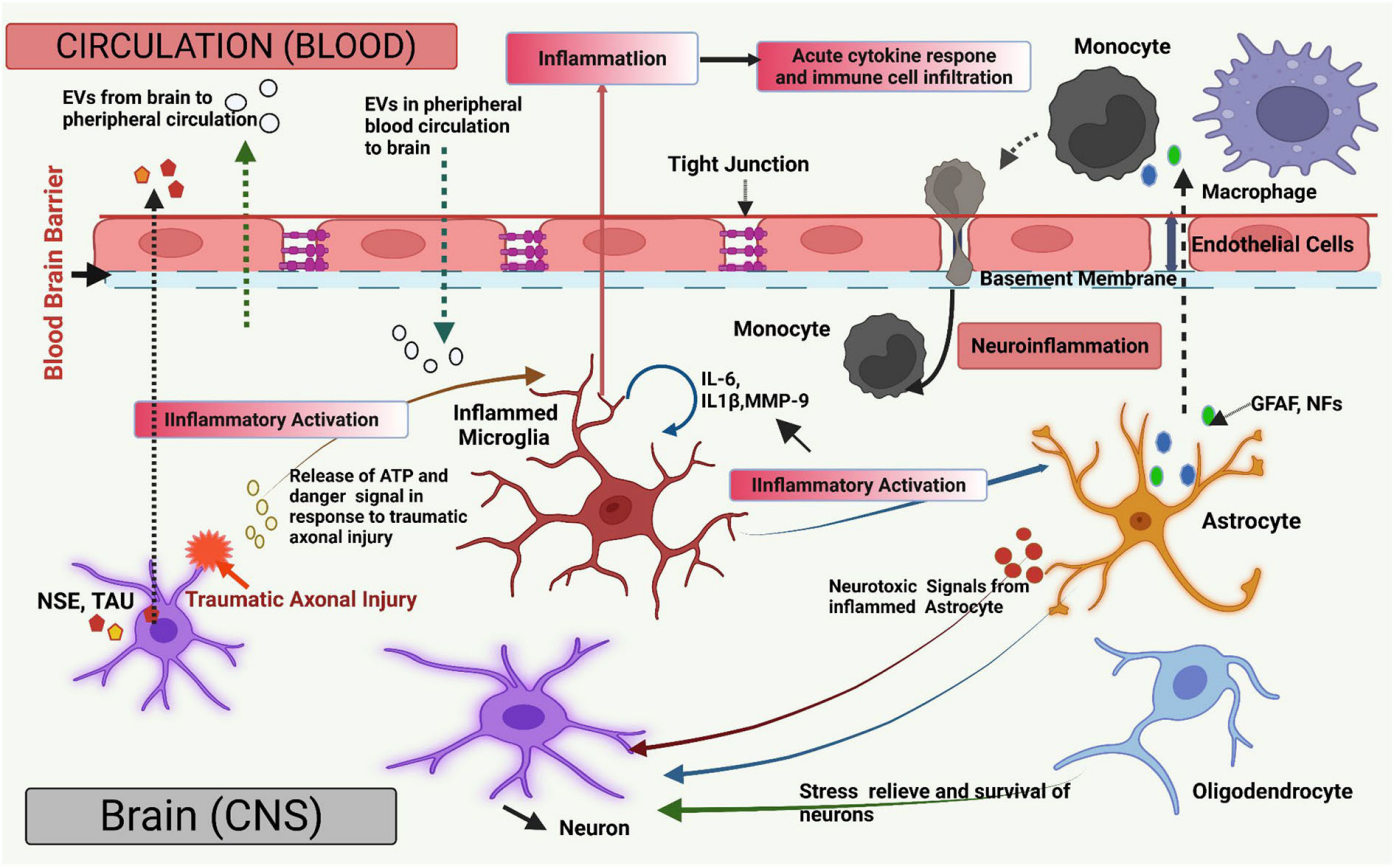
Overview of the extracellular vesicles (EVs) in brain injury. The diagram illustrates a graphical overview of the EV-mediated pathways and mechanisms that contribute to neuroinflammation and CNS damage (such as Traumatic brain injury; TBI) and are engaged in bidirectional cellular communication *via* EVs. All major types of CNS cells can send and receive EVs together with their molecular payloads, such as nucleic acids, functional proteins, and lipids molecules. Neuronal insult causes the release of ATP and molecular danger stimulus, which can produce pro-inflammatory EVs by microglia (IL-6, IL-1β). Microglial-derived EVs transmit inflammation *via* pro-inflammatory stimulation of other microglia and astrocytes. Microglial pro-inflammatory EVs can transverse the blood–brain barrier (BBB) to communicate with peripheral receptors upon neuronal absorption. After being stimulated with pro-inflammatory cytokines, astrocytes and endothelial cells generate EVs that cross the BBB and spread neuroinflammation by inducing the acute pro-inflammatory cytokine response, enabling molecular crosstalk between brain cells in the peripheral circulation. EVs, associated with inflamed astrocytes, generate neurotoxic chemicals. Also, EV-associated Astrocyte (Glial Fibrillary acidic protein; GFAP, Neurofilaments; NFs) and neuron-specific proteins (Neuron-specific enolase; NSE, TAU) may be released from damaged neurons due to traumatic axonal injury and enter the blood if BBB integrity is compromised, and their presence may indicate neuronal injury. EVs generated from oligodendrocytes and astrocytes transfer prior protein to a neuron which aids in neuronal stress relief and survival under ischemic and hypoxic conditions. EVs are also involved in the propagation, dissemination, and clearance of abnormal and neurotoxic proteins in neurodegenerative diseases. Figure created with BioRender.com.

All these findings imply that in NDs, the breakdown of the BBB allows EVs to go in both directions, from CNS to the periphery and vice versa. The disruption of the BBB is known to occur in many NDs, typically due to inflammation, providing another possible avenue for EV transport to the periphery. An assessment of connections between the systemic immune system and the CNS indicated that EVs facilitate mRNA transfer from hematopoietic cells to Purkinje cells of the cerebellum, altering gene expression in these cells, which indicates their functional significance (Ridder et al., 2014). EVs have also been transferred across the BBB using modified vesicles with a surface-protein-coated membrane to facilitate the transfer (Alvarez-Erviti et al., 2011). Endothelial cells that form and contribute to the BBB are also involved in the production of EVs, which transport proteins and play an important role in mediating intercellular signaling (Mazzucco et al., 2022). These EVs might be a source of NDs biomarkers, particularly in diseases involving BBB breakdown. Examination of EVs in serum samples by proteomics analysis has also uncovered the manifestation of CNS-specific proteins, indicating the presence of neuronal EVs in the blood of patients with Parkinson's disease (Ouerdane et al., 2022). Understanding how distinct EV subpopulations cross the BBB and interact to modulate barrier characteristics might develop therapeutic EVs for the CNS and boost the therapeutic potential of EVs-derived biomarkers for neurological diseases using peripheral biofluids (Figure 1).

## Insights and current perspectives on EVs and neurodegenerative disorders

NDs are one of the leading causes of mortality and disability and a significant financial strain on healthcare organizations (Luarte et al., 2016). EVs are considered to contribute to the etiology of NDs and play an important role in critical physiological activities of the CNS. EVs have the potential to serve as both diagnostic markers and therapeutic agents for TBI and NDs. NDs are characterized by the accumulation of toxic protein aggregates, which cause neuronal degeneration and death. Finding out how the disease alters the biochemical structure, properties, biogenesis, cargo composition, and intracellular communication of exosomes with their target cells will shed information on their function in disease regulation and, more crucially, their potential to serve as an ideal candidate for disease biomarkers. In TBI, possible functions for EVs are only now being investigated. This section briefly explores the studies demonstrating that EVs have a role in NDs, shedding light on the importance of EVs as diagnostic and therapeutic biomarkers.

The mechanisms behind neurodegeneration are diverse, but one common feature is the formation of aggregation of toxic proteins, which nucleate and propagate like prion proteins, ultimately leading to neurodegeneration and damage (Bellingham et al., 2012b). The toxicity might be attributed to multiple processes, including protein aggregation, mitochondrial dysfunction, axonal support disruption, synaptic network proteins toxicity, and stress to the endoplasmic reticulum membrane.

EVs are also involved in the local and long-distance transmission of neuronal-derived tau protein and a variety of mechanisms linked with the etiology of Alzheimer's disease (AD) (DeLeo and Ikezu, 2018; Badhwar and Haqqani, 2020). EV is also a potential candidate for biomarkers of other NDs, including Parkinson's and Creutzfeldt–Jakob disease (Bellingham et al., 2012b). EVs have also been associated with proteins, such as α-synuclein (Emmanouilidou et al., 2010) tau (Saman et al., 2012), and prions (DeLeo and Ikezu, 2018; Nisbet and Götz, 2018). EVs may play a part in the dissemination of amyloid proteins throughout the brain and in the production of seeds for harmful amyloid proteins in NDs. They act as a primary vehicle that transports amyloids out of cells and contributes to plaque development (Rajendran et al., 2014). Exosomes have been demonstrated to release amyloids in Alzheimer's disease, such as amyloid (A), and exosome-associated amyloids which can function as seeds for plaque development in the brain thereby, suggesting their role in the pathogenesis of AD (Rajendran et al., 2006).

EVs have been also reported to allow discrimination between stroke patients and controls and, to a lesser extent, the capacity to appropriately categorize the various stroke types. It has been shown that different types of cells including neural cells, endothelial cells, platelets, blood and vascular cells, and granulocytes release EVs in brain and circulation in acute phase of stroke (Jung et al., 2009; Kuriyama et al., 2010; Chiva-Blanch et al., 2016; Stenz et al., 2020). Moreover, Stroke-specific miRNAs generated from EVs have been described to be differentially expressed in both acute and subacute phases (Ji et al., 2016; Zhou et al., 2018; Wang W. et al., 2018). Table 2 outlines the studies related to candidate EVs biomarkers for NDs from different body fluids.

**Table 2:** The study of candidate EVs biomarkers for neurodegenerative diseases (NDs) from different biofluids.

**NDs**	**EVs source**	**Biomarkers**	**Expression**	**References**
**Alzheimer's disease (AD)**	Plasma-derived exosomes	p-t181-tau, Aβ-42	Upregulated	Fiandaca et al., 2015; Winston et al., 2016
	Plasma-derived exosomes & CSF	Aβ and NFT	Upregulated	Wang J. K. et al., 2017; Xiao et al., 2017
	Plasma-derived exosomes	NRGN	Downregulated	Winston et al., 2016
	Plasma-derived exosomes	HSF-1,LAMP-1, IRS-1 P-IRS-1,	Upregulated	Kapogiannis et al., 2015; Pluta and Ułamek-Kozioł, 2019
	Plasma-derived exosomes	Cathepsin-D, LAMP-1, Neurexin 2α, GluA4-containing glutamate receptor, NLGN1, NPTX2	Upregulated Downregulated	Goetzl et al., 2015b, 2018
	Plasma-derived exosomes	Heat Shock Protein-70	Downregulated in early and middle stages and upregulated in late stages	Goetzl et al., 2015b
	Serum-derived exosomes	SNAP-25	Downregulated	Agliardi et al., 2019
	Serum-derived exosomes	miR-135a, miR-384 and miR-193-b	Downregulated	Goetzl et al., 2015a
	CSF exosomes	miR-193b	Upregulated	Liu et al., 2014
	Serum-derived exosomes	MiR-342-3p,miR-342-5p	Collectively altered (Downregulated)	Lugli et al., 2015
	Plasma-derived exosomes	miR-150-3p,miR-185-3p, miR-338-3p, miR-342-3p,miR-332-5p,miR-24-3p, miR-23b-3p,miR-29b-3p	Differential expression	Lugli et al., 2015
	Serum-derived exosomes	hsa-miR-101-3p, miR-1306,hsamiR-106b	Upregulated Downregulated	Cheng et al., 2015
**Parkinson's disease**	CSF-derived exosomes	α-Synuclein	Upregulated	Stuendl et al., 2016
	Plasma-derived exosomes	α-Synuclein	Upregulated	Shi et al., 2014
	Plasma-derived exosomes	tau	Upregulated	Shi et al., 2014
	Salivary EVs	α-Synuclein	Upregulated	Cao et al., 2019
	Serum-derived exosomes	miRNAs, hsa-miR-374a-5p, hsa-miR-374b-5p, hsa-miR-199a-3p, hsa-miR-28-5p, hsa-miR-22-5p and hsa-miR-151a-5p	Upregulated	He et al., 2021
	Urinary exosomes	DJ-1	Upregulated	Ho et al., 2014
	Urinary exosomes	Ser(p)1292, LRRK2	Upregulated	Fraser et al., 2016

AD, Alzheimer's Disease; Aβ-42, Amyloid beta-42; CSF, Cerebrospinal fluid; NFT, neurofibrillary tangles; NRGN, Neurogranin; HSF-1, Heat Shock Factor 1; IRS-1, Insulin receptor substrate 1; P-IRS-1, phospho (P)-serine-type 1 insulin receptor substrate; LAMP-1, Lysosome-associated membrane protein; NPTX2, Neuronal pentraxin; NRXN2α, neurexin 2α; NLGN1, neuroligin 1; SNAP-25, synaptosomal-associated-protein-25; hsa-miR, human micro RNA; LRRK2, Leucine-rich repeat kinase 2.

## EV research in traumatic brain injury

TBI is a major cause of mortality and disability worldwide and a significant public health burden (El-Menyar et al., 2017; Quaglio et al., 2017). TBI can be caused by a blow or a penetrating injury to the head that causes mild to severe brain damage and affects normal neurological function (Khellaf et al., 2019). TBI is a complicated, heterogeneous, and debilitating disorder, especially among young people, with severe long-term consequences for survivors (Maas et al., 2017). The severity of TBI varies from mild–moderate to severe and is determined by clinical criteria, such as the occurrence and duration of loss of consciousness, memory loss, and changes in the mental status post-injury (Khellaf et al., 2019). TBI is widely recognized as a long-term illness that might have protracted health complications, and individual vulnerability to neurodegenerative alterations and continual symptoms remains largely unknown (Mollayeva et al., 2018). Lifestyle, gender, genetic and socioeconomic variables, and medical history, including past head injuries, are major TBI recovery determinants (Mac Donald et al., 2017).

Mild TBI (mTBI) is the most common kind of brain injury, impacting individuals of all ages (Silverberg et al., 2020). TBI can cause neurodegenerative alterations in milder cases, putting survivors at risk of acquiring long-term neurological and psychological issues. Multiple TBIs in populations, such as military personnel and contact sports players, have been associated with worsening neurobehavioral symptoms and poor outcomes (Rao et al., 2017; Pattinson et al., 2020). The complex nature of TBI, compounded with inadequate scientific knowledge underpinning disease pathophysiology, poses a challenge to developing successful and effective therapeutic modalities. Consequently, the consideration of modalities for accurate diagnosis and prognosis in TBI has spawned a number of studies to identify potential biomarkers to guide better clinical therapeutic strategies and identify individuals most at risk for poor recuperation and long-term repercussions (Gill et al., 2018a; Wang K. K. et al., 2018; Nitta et al., 2019).

## TBI as a risk factor for neurodegenerative disorders

The aberrant aggregation, accumulation, and deposition of protein in the brain are a shared component between traumatic brain injury (TBI) and several neurodegenerative illnesses. Some studies showed that TBI, regardless of severity, is a risk factor for different NDs (Fleminger et al., 2003; Rugbjerg et al., 2008; Jafari et al., 2013; Li et al., 2017). However, up to date, the relationship between TBI and NDsis inconsistent and remains a hot topic for researchers and clinicians (Huang et al., 2018a). The neuroinflammatory response after acute brain injury involves the release of pro- and anti-inflammatory cytokines, neurotrophic factor modulation, and cell migration to clear the resultant debris and heal the damaged region are tightly regulated and followed with a repair of the structural damage (Brett et al., 2022). However, this healing process is limited to the persistence of neuroinflammatory processes in some patients.

## Extracellular vesicles and TBI

TBI produces a variety of cognitive, bodily, and sensory symptoms as a result of acute injury-induced tissue damage and subsequent cellular and biochemical processes (Beard et al., 2020). EVs, especially exosomes, have garnered great excitement as potential novel diagnostic and therapeutic tools for TBI. EVs derived from the neuronal cells of CNS facilitate neuronal–glial cell communication, regulate neuroinflammation, and promote the dissemination of toxic proteins such as tau, and amyloid β, all of which collectively drive neurodegeneration in TBI (Frühbeis et al., 2013b; Kumar et al., 2017).

TBI, in most cases, is diagnosed by a neurological assessment of the patient, in combination with imaging modalities readily available such as computed tomography (CT) and magnetic resonance imaging (MRI) in clinical settings (De Guzman and Ament, 2017). The present clinical categorization modalities for TBI do not fully cover the disease's underlying etiology. Therefore, treating TBI properly using these clinical criteria alone is difficult. For example, the Glasgow Coma Scale (GCS) is commonly used for measuring functional outcomes by evaluating the level of patient consciousness. A total score of 13–15 denotes mTBI, 9–12 denotes moderate TBI, and 3–8 denotes severe TBI (Teasdale et al., 2014). However, these categorizations are contested due to inadequate descriptions of TBI variability (Mac Donald et al., 2017). The GCS score, for example, has limitations in diagnosing mild TBI in the context of multiple traumas, alcohol abuse, sedative use, and emotional stress (Di Pietro et al., 2017). Due to insufficient sensitivity and a lack of bleeding, standard imaging techniques such as CT and MRI scans generally fail to reveal lesions induced by traumas, which make characterization and prognosis of severe TBI relatively easy but recognizing mild and moderate TBI challenging (Silverberg et al., 2020).

Furthermore, imaging is widely utilized to identify brain lesions and abnormalities to determine the severity and localization of the injury for possible surgical planning (Smith et al., 2019). However, traditional imaging methods provide little assistance for therapeutic therapy. Mild TBI poses the most complex and unique diagnostic problem because it frequently contains microscopic injury involving axonal and vascular damages that alter biochemical, metabolic, and cellular homeostasis. Damage at the microscopic level may contribute to the development of long-term neurological impairments in the form of post-concussion complications (Marshall and Riechers, 2012).

A lack of specific biomarkers for TBI has been a significant impediment to the improvement of diagnostic assessment and therapeutic therapy (Bogoslovsky et al., 2016; Zetterberg and Blennow, 2016). TBI may compromise the structural integrity of the brain, leading to tissue, and vascular damage, intracerebral hemorrhage, and axonal shredding. As soon as the initial shock subsides, a complex chain of biochemical reactions linked with secondary injuries, such as alteration of the BBB, neuroinflammation, increased neuronal stimulation, and oxidative stress, begins (Masel and Dewitt, 2010; Blennow et al., 2012). These processes can typically last for longer periods after an injury, triggering regenerative and degenerative tissue responses (Blennow et al., 2012). The heterogenous and complex nature of TBI, along with a lack of knowledge of underlying pathophysiology, makes the development of effective therapeutic modalities very difficult. As a result, major hurdles remain in establishing definite effective therapies, diagnosis, prognosis, and improved stratification and characterization of TBI patients in order to optimize management and therapy approaches (Yue et al., 2020; Mondello et al., 2021).

Currently, despite significant efforts and the identification of a number of promising markers of acute and chronic head injury (Thelin et al., 2017; Diaz-Pacheco et al., 2022), not a single blood-based marker has been accepted or being used widely in the routine clinical practice (Mondello et al., 2021; Rogan et al., 2022). Therefore, the scientific community continues to search for innovative, more accurate, and refined TBI biomarkers. Several studies have sought to find biomarkers to guide treatments and identify people at the highest risk of poor recovery and persistent sequelae (Gill et al., 2018a; Nitta et al., 2019; Czeiter et al., 2020; Shahim et al., 2020). Several research groups have investigated candidate biomarkers detected in serum, plasma, and other body fluids (Sharma et al., 2017; Wang K. K. et al., 2018; McBride et al., 2022; Meier et al., 2022; Visser et al., 2022). The majority of investigations have concentrated on neuronal and astrocyte-derived proteins (Laverse et al., 2020; Meier et al., 2020; Mondello et al., 2020; Flynn et al., 2021; Garland et al., 2021; Richard et al., 2021). Other potential biomarkers include markers for neuroinflammation and vascular damage (Chiaretti et al., 2008; Li et al., 2016; Nitta et al., 2019) as well as circulating miRNAs that play important roles in gene regulation (Bhomia et al., 2016; Lafourcade et al., 2016; Di Pietro et al., 2017, 2018; Martinez and Peplow, 2017; Mitra et al., 2017, 2022; Qin et al., 2018; Das Gupta et al., 2021).

The long history of unsuccessful TBI-related clinical trials (Maas et al., 2013; Lener et al., 2015; Horton et al., 2018) has investigated innovative therapeutic strategies. Accumulating evidence suggests that EVs/exosomes regulate neuroinflammation, synaptic plasticity, and neurovascular integrity and alter BBB permeability, thereby controlling the cellular responses to brain damage (Lafourcade et al., 2016). There is strong evidence that EVs can arise in cells of the central nervous system (Pascual et al., 2020; Guedes et al., 2021; Schnatz et al., 2021) and play a critical role in regulating and modifying neuroinflammation, neuronal regeneration, and neurite outgrowth (Wang et al., 2011; Li H. et al., 2019). EVs exhibit several features that make them effective potential biomarkers of TBI. EVs' inadequate immune response, stability, long half-life, and capacity to transverse the BBB (Delpech et al., 2019) render the man excellent candidate for delivering therapeutic agents. As a result, exosomes (EVs) can be used as a liquid biopsy and as an alternative to imaging techniques. Blood-derived EVs are minimally invasive and can measure the biochemical and molecular alterations that occur in neuronal and glial cells of the brain following TBI. Almost all brain cells generate EVs that contain molecular payloads/cargos resembling their biogenic cells and are protected from damage when crossing the BBB (Wu et al., 2021). Thus, isolating EVs from different brain cell types and subsequently evaluating their unique cargo should increase our capacity to identify distinct TBI signature phenotypes, such as neuroinflammation, axonal damage, and neurodegeneration.

Moreover, biomarkers based on EVs may easily cross the BBB with their intact molecular payloads, which remain in circulation for a longer time (Witcher et al., 2015; Sulhan et al., 2020; Wu et al., 2021). This might be especially useful for diagnosing mTBI and could be an alternative to neuroimaging modalities for assessing TBI across different severities. Unlike surrogate protein indicators of acute TBI pathology, blood-derived EVs actively drive homeostatic and pathogenic processes as well as healing in the wounded CNS throughout the process of disease development (Frühbeis et al., 2013b).

Despite significant and rapid technological advancement, the study of EVs population in brain injury is an emerging field. The intrinsic difficulty in the differential diagnosis of head traumas is well established. The variability of clinical presentation, the large number of unreported injuries, and the various etiologies inherent in the injuries considerably raise the chance of misinterpretation. Most current proteomic techniques focus on analyzing the small number of neuronal damage indicators that are currently known, either single or in pairs. Furthermore, numerous research projects have focused on detecting these indicators in the CSF. Although these techniques are viable, they pose significant clinical hurdles due to the difficulty of obtaining the CSF sample, which requires an intrusive procedure. Identifying trauma-specific biomarkers in a peripheral blood sample might serve as a de facto “liquid biopsy” for concussion or TBI, considerably benefiting doctors in the differential diagnosis and evaluation of the brain injury (Rayyan et al., 2018).

## EVs and inflammation in TBI

Neuroinflammation is recognized to have a significant role in the pathophysiology of TBI by exacerbating the secondary injury (Woodcock and Morganti-Kossmann, 2013; Lozano et al., 2015; Sulhan et al., 2020). The acute TBI leads to primary brain insult. Moreover, insight into the etiology of traumatic brain injury has revealed that hyper neuroinflammation, disruption of the BBB, oxidative stress, mitochondrial alteration, and disruption of synaptic plasticity and neurovascular integrity lead to disruption of the activation of downstream secondary injury cascades (Sulhan et al., 2020). The very first inflammatory response is initiated to defend the injured region against invading germs and tissue waste containing toxins. However, the bulk of secondary cell death following TBI is caused by the overactivation of neuroinflammation, which includes microglia, astrocytes, inflammatory mediators such as cytokines and chemokines, and other invading immune cells (Sulhan et al., 2020). In addition, TBI induces a complex cascade of systemic inflammatory responses that can persist for longer durations after the initial injury. Initial inflammation has been shown to have protective effects, such as clearing away cell and tissue debris and protecting against pathogens. On the other hand, prolonged neuroinflammation is hazardous because it can lead to TBI progression, worsening of the initial injury, neurodegeneration, and delayed cell death (Schimmel et al., 2017).

Recent translational investigations have shown that EVs can activate the immune system and promote inflammation by transporting and discharging a range of pro-inflammatory mediators (Zhao et al., 2017; Alam et al., 2020), thereby playing an important regulatory role in neuroinflammation in multiple neurological diseases (Khan et al., 2018; Sulhan et al., 2020). A recent seminal study investigated the function of microglial MVs in promoting inflammation of the brain in a mouse model (Kumar et al., 2017). The study showed that microglia-derived extracellular vehicles with high concentrations of pro-inflammatory molecules (such as IL-1 and miR-155) are discharged into the bloodstream following TBI. This increases the neuroinflammatory responses by activating microglial cells, leading to enhanced expression of pro-inflammatory molecules (Kumar et al., 2017). Another study found that exosomal miR-124-3p improved neurologic consequences and reduced inflammatory response to TBI by reducing microglia activation and mTOR signaling activity (Huang et al., 2018b).

Furthermore, exosomes promote neurite development *via* miR-124-3p translocation into neurons. These studies have shown that chronic inflammatory reactions are key contributors to the development of NDs. Understanding ways to reduce and modify neuroinflammation following TBI and possible therapeutic applications remains a significant research priority domain.

## EVs as diagnostic and therapeutic conduits in TBI: Examining TBI with EV-based biomarkers

### Extracellular vesicle miRNAs

Micro RNAs (miRNAs) are small, single-stranded, non-coding RNA molecules (containing ~22 nucleotides), which are involved in the post-transcriptional regulation of genes in eukaryotic cells (Towler et al., 2015). Micro RNAs have sparked attention as potential biomarkers and therapeutic targets in TBI. A multidimensional Exo-proteomic approach involving EVs has shifted the attention from traditional markers of brain injury toward a targeted, personalized evaluation of TBI and its progression and recovery trajectories.

A recent study looked at miRNAs isolated from plasma, and plasma-derived EVs from military veterans with mTBI and 45 and 32 miRNAs were shown to be differentially regulated in EVs and plasma, respectively (Ghai et al., 2020). Neuroinflammation, vascular modeling, and function of neuronal cells have been shown to be related to differently regulated miRNAs (Ghai et al., 2020). Additional research has identified a biomarker panel based on miRNAs that can identify TBI in animal models and humans (Ko et al., 2018, 2020). Using microRNA sequencing of GluR2+ extracellular vesicles across various injury severity, types, and time frames, another study has discovered discrete TBI profiles across numerous models of injury and post-trauma periods, as well as panels of biomarkers capable of detecting, and identifying specific phases of injury (Ko et al., 2020). This work demonstrated that in a mouse model of mild to moderate TBI, neuronal-derived EVs exhibited varying expression levels of miRNAs involved in regulating multiple distinct pathways, including stimulatory effects, neurotransmitter signaling, and intracellular pathways (Ko et al., 2020).

A study by Harrison et al. investigated the miRNA cargo of neuronal-derived EVs isolated from brain injury models of mice and controls to evaluate the relevance of EV-associated miRNA in TBI (Harrison et al., 2016). They observed that miR-146, miR-7a, miR-21, and miR-7b expression increased considerably, with miR-21 showing the highest variation among conditions. Micro RNA-21 (MiR-21) was shown to be released from neurons as probable EV cargo, as evidenced by the simultaneous increase in miR-21 in EVs and neurons. This study reported a novel cell–cell communication pathway in TBI (Harrison et al., 2016). Studies examining the role of EVs associated biomarkers of TBI in animals and humans are listed in Table 3.

**Table 3:** Studies related to EVs as biomarkers in TBI.

**EVs**	**Study type**	**Biomarkers**	**Origin of biomarkers**	**Observations**	**Isolation and characterization method**	**References**
Microvesicles (MVs)	*In vivo* and *in vitro* both	81 miRNA molecules in CSF microparticles. (e.g., miR-9 and miR-451)	CSF derived MVs	MPs were found to be augmented in CSF following severe TBI	Serial centrifugation • Ultracentrifugation • Flow cytometry, • Electron microscopy, • Polymerase chain reaction (PCR),• Western blotting	Patz et al., 2013
Microvesicles	Humans *In vivo*	Tissue factor (TF) and P-selectin	Endothelial platelet and leukocyte-derived MVs	After a severe TBI, the number of MPs was augmented	Serial centrifugation and Flow Cytometry	Nekludov et al., 2014
	*In vivo* (Rat model of TBI)	Not reported	Exosomes derived from MSCs	Cell-free MSC-generated exosomes improved functional recovery after TBI in rats by enhancing endogenous angiogenesis and neural regeneration while reducing inflammation.	qNano,• Transmission electron• microscopy, Western blot	Zhang et al., 2015
Microvesicles	*In vivo* (Mice)	Tight junction proteins (TJPs)	Microvesicles derived from brain endothelium	Following a TBI, the cerebral endothelium undergoes vascular remodeling *via* the release of EVs carrying tight junction proteins and endothelial markers.	Exoquick Flow cytometry,• Electron microscopy,• Western blotting	Andrews et al., 2016
Microvesicles	*In vivo* (Mice)	miRNA (miR-21, miR146, miR-7a, and miR-7b)	EVs derived from the neuronal cells	Increased expression of miR-21, miR-146, miR-7a, and miR-7b in EVs and decreased expression of miR-212 in the brain of mice model of TBI	Repeated centrifugation and• Electron microscopy,• Sequencing of miRNA	Harrison et al., 2016
Microvesicles	*In vivo*	Glial fibrillary acidic protein (GFAP), neuron-specific enolase (NSE), and aquaporin-4 (AQP4),	Neuronal–glial-derived Microparticles	When compared to healthy controls, the severe TBI group had larger concentrations of MPs expressing GFP and AQP4.	Serial centrifugation• flow cytometry	Nekludov et al., 2017
Exosomes	*In vivo* (Severe TBI patients)	(NOD)-like receptor protein-1(NLRP-1) inflammasome proteins	Peripheral blood, CSF-derived exosomes	CSF-derived exosomes showed Increased expression of inflammasome proteins in TBI patients – Exosomes derived from neurons deliver siRNA (short interfering RNA) into the CNS to inhibit inflammasome activity.	ExoQuick method Western blot	De Rivero Vaccari et al., 2016
Microvesicles	*In vivo* (Mice)	Pro-inflammatory cytokines, interleukin-1β and miR-155	Microglial derived microvesicles	Microglial-derived MPs contributed to developing and disseminating neuroinflammation following TBI by stimulating microglia and increasing systemic immune responses.	Serial Ultracentrifugation Flow cytometry	Kumar et al., 2017
Exosomes	*In vivo* (Human)	Tau protein, amyloid-beta (Aβ42), and cytokines (tumor necrosis factor-alpha (TNFα, interleukin (IL)-6 and−10)	Neuronal derived exosomes	Concentrations of exosomal tau, Aβ42, and IL-10 were elevated in the mild TBI group suggesting that central inflammatory activity contributes to PTSD symptoms	Ultracentrifugation,• ExoQuick and digital array technology by single-molecule enzyme-linked immunoassays (Simoa™)	Gill et al., 2018b
Exosomes	*In vitro* *In vivo* (Mice)	miR-124-3p	Microglial derived exosomes	MiR-124-3p levels in microglial exosomes increase from acute to chronic stages of TBI/miR-124-3p transfer in microglia through exosomes decreasing neuronal inflammation and promoting neurite outgrowth *in vitro*, enhancing neurologic outcome and suppressing neuroinflammation *in vivo*.	Centrifugation• Electron microscopy NanoParticle Tracking,• miRNA microarray analysis	Huang et al., 2018b
Exosomes	*In vivo* (Rat)	human adipose mesenchymal stem cell (hADSC)-derived exosomes (hADSC-ex)	Mesenchymal stem cell-derived exosomes	Intracerebroventricular microinjection of human adipose mesenchymal stem cell-derived exosomes (hADSC-ex) inhibited neuroinflammation, decreased neuronal death, and enhanced neuronal regeneration in the rat model of TBI.	Flow cytometry,• western blot, ELISA, qRT-PCR	Chen et al., 2020
Exosomes	*In vivo* (Human)	miR-873a-5p	Astrocyte-derived exosomes	By inhibiting the NF-B signaling pathway, miR-873a-5p, reduced microglia-mediated neuroinflammation and ameliorated cognitive deficits following TBI	Immunofluorescence, Western blot Electron microscopy	Long et al., 2020
Exosomes	*In vivo* (Rat model of TBI) *In vitro*	miR-216a-5p	Exosomes derived from mesenchymal stem cells	Brain-derived neurotrophic factor (BDNF)-BDNF-induced MSCs-Exosomes successfully inhibited inflammation and promoted neuronal regeneration	Hyper centrifugation and Western blot Transmission electron microscopy (TEM)	Xu et al., 2020
Exosomes	*In vivo* (Rat model of TBI) *In vitro*	miR-17-92	Multi-potent mesenchymal stromal cell (MSC)-derived exosomes	Exosomes enriched in the miR-17-92 cluster enhance functional recovery following TBI by reducing neuroinflammation and increasing endogenous angiogenesis and neuronal regeneration	Western Blot, qRT-PCR Immunofluorescence	Zhang et al., 2021

These investigations highlight the vast range of EVs molecular cargo expressed in readily available samples of EVs, which might give a plethora of biochemical information for evaluating differential diagnosis, and therapeutic routes involved in TBI.

### Blood-based EVs as biomarker reservoirs for different severities of TBI

The complex nature of TBI, compounded with inadequate scientific knowledge underpinning disease pathophysiology, poses a challenge to developing accurate diagnostic, prognostic, molecular biomarkers, and effective therapeutic modalities. The quantification of EVs as a biomarker has been investigated in studies using serum and plasma-derived EVs without sample enrichment for specific EV subtypes (Younas et al., 2022). The majority of attempts to establish EV-derived biomarkers of TBI have centered on leveraging the presence of traditionally investigated blood-derived proteins inside EVs for diagnosis and evaluating neurological impairments predicting the outcome (Winston et al., 2019; Vaughn et al., 2021).

Mild TBIs (mTBI), which include concussions, are the most common TBI resulting in post-TBI survival (Lefevre-Dognin et al., 2021). Although mTBI may cause neuropathological alterations, the early clinical symptoms are primarily due to functional impairment rather than structural damage (Sussman et al., 2018). As a result, abnormalities are seldom seen with conventional structural neuroimaging, and mTBI are frequently undiagnosed since traditional techniques, i.e., computed tomography (CT) and magnetic resonance imaging (MRI), may not be able to detect micro-lesions or injuries (Shin et al., 2017).

In addition to CSF-based biomarkers, a number of promising blood-based TBI biomarkers have been reported (Azar et al., 2017; Edalatfar et al., 2021; Hvingelby et al., 2022). However, because of the restrictions in their diffusion over the BBB, blood levels of free circulating proteins and mRNA are readily degraded and can fall below detection levels for identification. Furthermore, the provenance of the tissue is unclear, limiting the interpretation of potential mechanistic contributions (Ghaith et al., 2022). As a result, there is an unmet clinical need for accurate biomarkers that can identify all TBIs, including mTBI, and predict the likelihood of developing long-term sequelae, including post-concussive syndrome chronic traumatic encephalopathy (Ghaith et al., 2022; Mavroudis et al., 2022). Recent research has revealed that circulating exosomes enriched for CNS-specific tissue sources may be a method to develop practical biomarkers for TBI, particularly mild TBI, to circumvent these difficulties (Goetzl et al., 2019, 2020; Winston et al., 2019; Vaughn et al., 2021).

The microtubule-associated tau protein is mainly expressed in the neurons, where they stabilize microtubules in axons (Barbier et al., 2019; Li et al., 2022). Normally, tau undergoes phosphorylation to regulate the movement of microtubules. When they are hyperphosphorylated, tau accumulates and forms neurofibrillary tangles. These tangles disrupt neuronal functions and induce neurodegenerative changes in the brain (Alonso et al., 1996; Barbier et al., 2019). Several studies have found links between total tau protein or phosphorylated tau and mild, moderate to severe TBI incidents applying CNS-derived exosome enrichment methods. Despite the variability and heterogenicity in TBI presentations, exosomal total tau has been found to be augmented in mild, moderate, severe, and repetitive TBI patients from days to years after the initial injury (Stern et al., 2016; Kenney et al., 2018; Gill et al., 2018b; Goetzl et al., 2019, 2020; Muraoka et al., 2019, 2021; Mondello et al., 2020), suggesting it to be relatively sensitive marker which exhibits both an acute and a chronic temporal course.

Aβ42 protein is derived from the amyloid precursor protein, which is normally located in the synaptic membranes of neurons (Sohma, 2016; Chen G. F. et al., 2017). Cleavage in the amyloid precursor protein can result in the accumulation of Aβ isoforms like Aβ42 and the subsequent development of brain plaques (Sohma, 2016). Accumulating evidence suggests that Aβ plaques have a role in neurotoxicity and the development of TBI (Johnson et al., 2010; Bird et al., 2016; Edwards and Soto, 2017). The build-up of Aβ42 in the soma and axon of neurons following TBI is a potential contributor to the persistence of neuronal impairment (Edwards and Soto, 2017). Multiple investigations, similar to those showing an increase in tau protein, have revealed that Aβ42 is upregulated and increased in isolated exosomes in every clinical classification of TBI (mild, moderate, severe, and repetitive). Moreover, Aβ42 has been found to be increased in both neuronal and astrocyte-enriched exosomal samples of TBI patients and remained in circulation from days to years after the initial injury (Edwards and Soto, 2017; Gill et al., 2018b; Goetzl et al., 2019, 2020; Winston et al., 2019; Vaughn et al., 2021).

Multiple studies examined the expression levels of neuronal and glial damage indicators in the exosome profiles of people with TBI. Neurofilament light chain (NFL), a structural scaffolding protein abundantly expressed in long myelinated axons, is one of the most studied biomarkers due to axonal injury caused by trauma (Lee et al., 2020). NFL levels in plasma-derived exosomes have been shown to be higher in mild, moderate, and severe TBI patients at different time points following damage compared to controls (Mondello et al., 2020; Peltz et al., 2020; Guedes et al., 2020b, 2021, 2022) indicating exosomal NFL measurements are detectable in TBI's acute and chronic course.

The cytoplasmic enzyme UCH-L1 (ubiquitin carboxy-terminal hydrolase L1) is essential for the preservation of axonal and neuronal health (Wang K. K. et al., 2017). Neuronal-derived exosomes have been reported to have UCH-LI elevated at an acute level (within 7 days) but not chronically in moderate TBI victims (Goetzl et al., 2019). In a study reporting temporal profile, the levels of UCH-L1 in total exosomes decreased after 24 h post-injury in patients with moderate to severe TBI (Mondello et al., 2020). Also, the median levels of total exosomal UCH-L1 were found to be elevated 8-fold higher as compared to day 5, indicating a significant reduction (Mondello et al., 2020).

Glial fibrillary acidic protein (GFAP) is a cytoarchitectural protein found in astrocytes. Its presence in the blood serum and CSF has frequently been used as a glial injury marker (Abdelhak et al., 2022). Therefore, the role of GFAP as a biomarker for neurodegeneration and acute astrocytic damage is being investigated. Studies have shown that exosomal levels of GFAP in moderate to severe TBI patients are significantly increased up to 48 h of initial injury (Mondello et al., 2020; Peltz et al., 2020; Flynn et al., 2021). Although GFAP levels decrease immediately following injury, persistent increases in GFAP have been linked to long-term cognitive damage in TBI veterans (Nekludov et al., 2017; Peltz et al., 2020; Puffer et al., 2020). However, a correlation or link between GFAP and mild or repeated TBI has not yet been established.

Aquaporins (AQPs) have been found to play a role in EVs and have emerged as a promising candidate to play an essential role in regulating the early stage of TBI. Aquaporin-4 (AQP4) is a water channel transmembrane protein widely distributed in the glial cells. It is crucial in regulating neuroinflammatory and edema processes in the brain (Liu et al., 2021; Dadgostar et al., 2022). Even though the role of EVs-based AQP4 in TBI has been established recently, it is already being suggested as a biomarker for disease, drug targets, and possible treatments of TBI. Studies have shown that total EV levels of AQP4 are significantly augmented in mild and severe TBI patients vs. those without injury (Nekludov et al., 2017; Dadgostar et al., 2022).

Furthermore, research on EV-based biomarkers has also concentrated on a condition known as chronic traumatic encephalopathy (CTE), one of the most devastating possible sequelae of TBI. CTE is characterized by a specific dispersion of tau protein pathology and can be produced by multiple mild TBI and a single severe TBI that does not elicit concussion (McKee et al., 2009). The levels of tau protein have been shown to be augmented in plasma and CSF specimens following acute TBI (Rubenstein et al., 2017). However, these proteins have not been widely acceptable biomarkers of CTE in clinical settings (Stern et al., 2016); it is being postulated that proteins associated with EVs may accurately evaluate neuronal damage following TBI.

In a study, Stern et al. (2016) observed that the concentrations of tau protein in plasma-derived EVs were higher in ex-football players who had mild recurrent TBIs (rTBIs) than controls in sports-related mTBI, indicating its possible utility as a predictive biomarker of CTE. Another study by Kenney et al. (2018) examined plasma-derived exosomal concentrations of amyloid-beta (A), tau protein, and phosphorylated tau (p-tau) in persons with a history of combat-related mTBI with persistent cognitive impairment symptoms. rTBI patients had augmented exosomal tau and p-tau levels than those with two or fewer mTBI and those without TBI. Exosomal tau and p-tau levels were shown to be substantially linked with post-traumatic and post-concussion effects, suggesting their potential value as a biomarker for mTBI prediction. Overall, these studies and the current research scenario employing EVs as potential biomarkers of TBI suggest that detecting a wide variety of brain-derived EV payload molecules may give an additional distinct perspective of the pathogenic mechanisms behind TBI sequelae.

### Microglial derived EVs in TBI

It is believed that past exposure to a series of rTBI renders the brain more sensitive to degenerative processes following a head injury, which may be regulated in part by neuronal–glial cells (Manley et al., 2017). Following a traumatic brain injury, microglial cells remain in an inflammatory state. Inflamed microglia have diminished ability and threshold for reacting to stimuli that influence the function of the brain (Witcher et al., 2015). Neuroinflammation is a hallmark in the brain with CTE, with a substantial elevation in the stimulated microglia cells in the brain's white matter (Gardner and Yaffe, 2015). Moreover, the activation of microglia may be advantageous in the initial stages of damage but can subsequently be deleterious. However, the significance of microglial EV miRNAs in controlling TBI neurodegeneration is yet unknown.

The investigation of microglial-derived extracellular vesicles (MDEs) miRNAs in a mouse model of rTBI showed that microRNA-124-3p had a neuroprotective effect on recovery trajectories in TBI by promoting the polarization of M2 in microglial cells and inhibiting neuroinflammation (Huang et al., 2018b). Similar to these observations, a study by Yang et al. (2019) revealed that treatment with EV-derived micro RNA (miR-124) boosted hippocampal regeneration by promoting the polarization of microglial M2 cells, which were achieved *via* blocking Toll-like receptor 4.

Another study by Li D. et al. (2019) reported that enhanced miR-124-3p in milk-derived exosomes (MDE) increased neurite development by arresting neuronal autophagy and providing protection against neuronal injury. In another investigation, the micro-RNA levels of MiR-124-3p in MDE have been shown to be significantly altered throughout the acute, subacute, and chronic phases of rTBI (Ge et al., 2020). Notably, the surface markers considered to recognize myeloid cells in the CNS are expressed by both microglial cells and macrophages (Depaula-Silva et al., 2019). The studies discussed above demonstrate that MDEs play a role in neurodegeneration and neuroinflammation; microglial EVs may be an effective and potential candidate biomarker in TBI.

## EVs as potential predictors of functional outcomes in TBI

Risks for post-traumatic stress disorder (PTSD), depression, and other mental diseases increase dramatically after a TBI (Barnes et al., 2018; Roy et al., 2019; Stein et al., 2019; Kulbe et al., 2022). Patients dealing with TBIs' long-term effects are often at the risk of developing cognitive impairment (CI) or post-concussion syndrome (PCS) (Mehrolhassani et al., 2020). Although EVs-based biomarkers can detect neurological injuries, their therapeutic relevance would be significantly enhanced if they could predict functional consequences, such as cognitive decline or mental health symptoms.

Cognitive impairment has been the most frequently studied functional outcome associated with exosome protein cargo in patients with TBI. A study studying the long-term effects of TBI, reported p-tau, cellular prion protein (PrPc), Aβ42, and synaptogyrin-3 to be elevated in plasma NDEs of TBI patients with CI but not in healthy controls (Goetzl et al., 2020). A separate study reported IL6, NFL, TNF-alpha, and GFAP to be elevated in NDEs of TBI patients with CI compared to TBI patients without CI providing evidence that elevated levels, CNS-enriched exosomal biomarkers linked with TBI and CI can be detected even decades after initial TBI (Peltz et al., 2020). Another preliminary study had shown that total exosomal tau levels significantly correlated with cognitive functioning as measured by short-term memory in retired professional football players (Stern et al., 2016). Studies involving proteomic analysis of CSF-derived and plasma-derived EVs from retired National football league (NFL) players who were at risk of developing CTE found a significant correlation between t-tau and p-tau181 levels in CSF, but not in a control group (Muraoka et al., 2019, 2021).

In addition to CI, several studies have also identified EVs cargo links with neuropsychiatric symptoms (Gill et al., 2018b). It has been reported that NDE tau and NFL markers are also associated with PCS symptoms and PTSD (Kenney et al., 2018; Pattinson et al., 2019; Guedes et al., 2020b, 2021, 2022). Some studies have also reported an increased NDE, and total exosomal levels of p-tau and total tau in patients with mild TBIs are associated with increased PTSD symptoms (Kenney et al., 2018; Gill et al., 2018b). However, most of these studies involved small sample sizes, making it challenging to generalize their results regarding functional outcomes in the cognitive and psychiatric domain, and there is no substantial clinical evidence yet, on how EVs cargo might forecast future cognitive impairment after TBI. To distinguish whether EVs are linked with TBI-specific cognitive impairment, longitudinal investigations with larger sample sizes will be sorely needed for clearer insight.

## MSC-derived exosome as a novel therapy for TBI

Over the last decade, scientific and clinical findings have backed cell therapies, such as bone marrow-derived mesenchymal stem cells (MSCs), which have shown promise as a viable treatment modality in variety of NDs, including TBI (Das et al., 2019). MSCs-derived exosomes may provide various benefits over traditional cell-based therapies, including superior safety profile, being less invasive, higher stability, simple transfer, and inducing minimal or no immune responses (Dehghanian et al., 2020). Neurological illnesses induce sensory and motor dysfunctions and anomalies in the CNS. Furthermore, damaged axons lack the ability to recover spontaneously due to a restrictive cellular environment, diminished neuronal cell regeneration capacity, and a lack of growth-promoting chemicals (Anderson et al., 2016). Surgical intervention and pharmaceutical therapy may reduce but not totally cure, making NDs treatment a critical issue in the clinical setting (Silberberg et al., 2015). Exosomes produced from MSCs may serve as a non-invasive intervention for the delivery of therapeutic drugs to the brain and may be useful in the treatment of TBI (Xiong et al., 2017). Therefore, it is important to investigate the newer treatment modalities for the elimination of neurological illnesses progression.

MSCs have been extensively employed in regenerative medicine for therapeutic purposes (Levy et al., 2019). MSC treatment for neurological illnesses has shown neuroprotective potential in both laboratory and clinical investigations (Levy et al., 2019; Li P. et al., 2019). MSCs are adult stem cells generated from mesoderm, which can self-renew and differentiate in several directions. They are found in different tissues and organs, including bone marrow (Zhang et al., 2015; Staff et al., 2019). MSCs-derived exosomes have also been shown to play critical functions in the paracrine route and exert neuroprotective properties and therapeutic efficacy by healing the damaged cellular-microenvironment (Staff et al., 2019; Zhang K. et al., 2022). There is compelling evidence that MSC-derived EVs may exert therapeutic effects following TBI in animal models (Xin et al., 2013a; Doeppner et al., 2015; Xiong et al., 2017).

Zhang et al. (2015) conducted a seminal work in which they found that intravenous administration of MSC-derived exosomes enhanced cognitive and sensory and motor functional outcomes in a rat model of traumatic brain injury. MSC-derived exosomes have also been reported to improve vascular integrity and density, promote angiogenesis and neuronal regeneration, and diminish neuroinflammation of the brain (Zhang et al., 2015), while having no effect on the volume of the cortical lesion. Similarly, another study showed that infusion of EVs isolated from human MSCs suppressed neuroinflammation and improved functional outcomes 1-month post-injury (Kim et al., 2016). Another important study demonstrated that MSC-derived exosomes, when administered intravenously, in a dose- and time-dependent manner, led to substantial neuroprotective and neurohealing effects by reducing loss of neuronal cells, neuroinflammation and augmenting neurogenesis and angiogenesis in a rat model TBI (Zhang et al., 2020). Accumulating evidence also suggests that human umbilical cord mesenchymal stem cells (HUCMSCs) aid neurological recovery following TBI. Moreover, exosomal HUCMSCs enhanced sensorimotor function and neurovascular remodeling, prevented apoptosis, and decreased neuroinflammation, leading to a significant recovery in functional outcomes in a rat model of TBI (Zhang Z. W. et al., 2022), indicating their potential as a viable and emerging therapeutic option for treatment of TBI.

Furthermore, research into the involvement of miRNAs in MSC-derived exosomes as potential neuroplasticity mediators might be an attractive field of study. It has been postulated that exosomes deliver miRNAs to the brain, promoting neuroplasticity and functional recovery following brain damage. Functional miRNAs, for example, delivered from MSCs to brain cells through exosomes have been shown to increase neurite rebuilding and functional recovery in stroke rats (Xin et al., 2013b). The above studies indicate that MSCs-derived exosomes can potentially act as a non-invasive intervention for the transportation of therapeutic agents into the brain and further be applied in treating TBI. Although prior research has yielded promising findings, we are only at the beginning of our understanding of the potential of MSC-derived exosomes as a feasible therapeutic strategy for TBI, and further investigation is necessary to ascertain the function of active exosomal miRNAs in fostering functional recovery and neurovascular remodeling, controlling neuroinflammation and peripheral immune response, and regulating brain growth factors.

**Figure 2:**
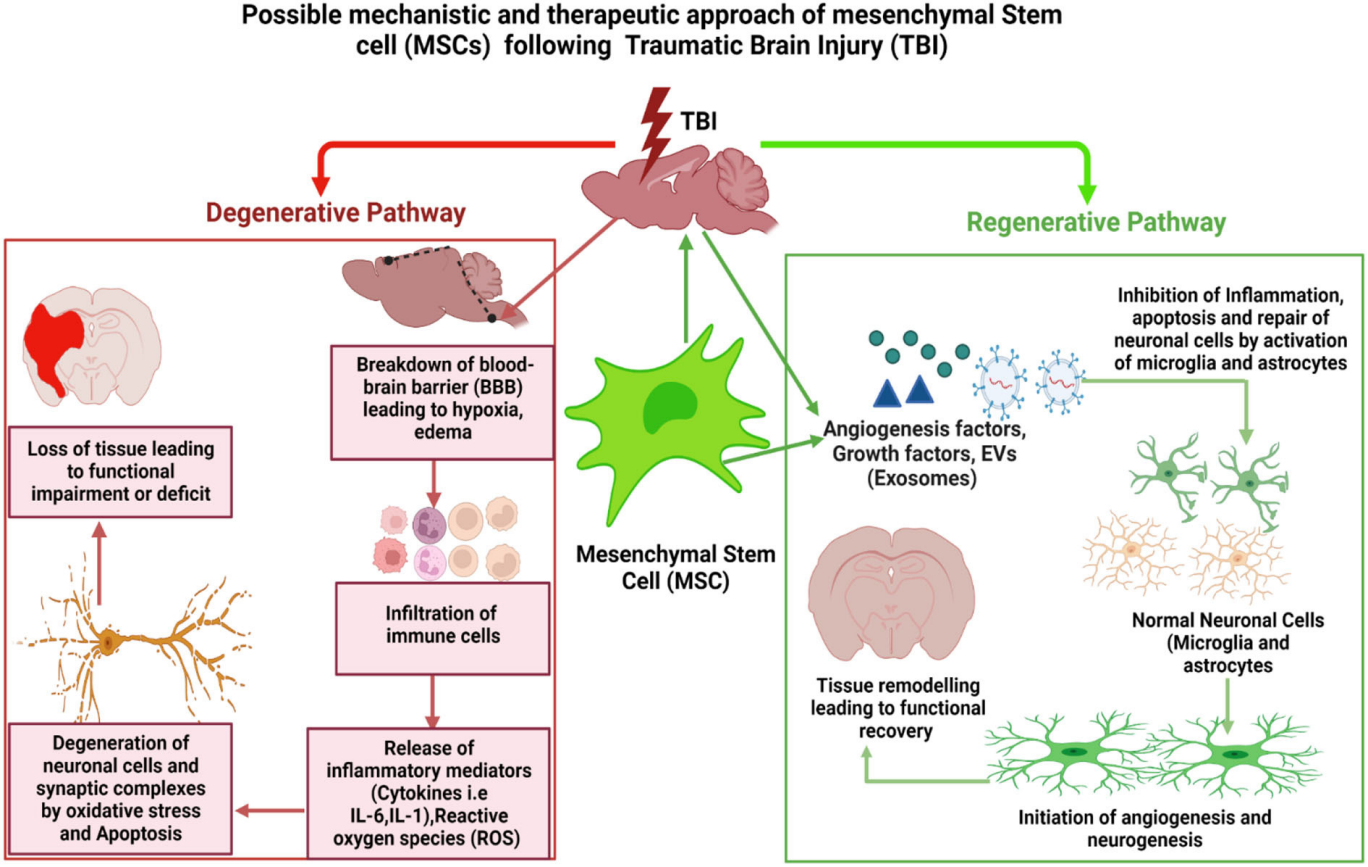
Possible mechanistic and therapeutic approach of mesenchymal stem cell (MSCs) following traumatic brain injury: TBI breaches the blood–brain barrier (BBB), resulting in a cascade of reactions such as hypoxia, edema, cellular infiltration, and release of inflammatory mediators by immune cell. This causes an increase in the formation of reactive oxygen species (ROS), which causes oxidative stress, neuroinflammation, and apoptosis, all of which damage neurons and activate astrocytes and microglia. Activated astrocytes and microglia set off inflammatory processes that further damage neurons and other cells, leading to tissue loss and functional deficiencies over the long term. When we use MSCs to treat traumatic brain injury (TBI), MSCs and their released exosomes can cross the blood–brain barrier (BBB) and migrate steadily to the damaged area of the brain. There, they release a variety of cytokines, including neurotrophic factors and vascular regeneration factors, to promote nerve and blood vessel repair and regeneration, leading to the restoration of the brain microvascular system and its function. Figure created with BioRender.com.

Taken together, these results show that MSC-derived exosomal treatment modality appears to be a viable and promising approach that might considerably enhance our understanding of the pathogenesis and neuroprotective mechanism involved in TBI. However, more extensive investigation is required with respect to the time and dosage-dependent efficacy, toxicity, and methodological considerations of MSC-derived exosome, which would ultimately determine their therapeutic utility in clinical settings. Figure 2 shows the possible mechanistic and therapeutic approach to mesenchymal stem cell (MSCs) following traumatic brain injury.

## Extracellular vesicles: Methodological concerns

The EVs have been discovered and isolated in different body fluids, such as peripheral blood, saliva, CSF, breast milk, and urine, making them easily available (Fauré et al., 2006; Yáñez-Mó et al., 2015; Kumari and Anji, 2022). Recently, the technological barrier of conclusively separating exosomes from MVs has been a major impediment to advancement in understanding and possible therapeutic use of EVs. This, of course, increases biases when describing EV features for molecular research. As a result, one of the field's major priorities is to enhance and standardize EV isolation and analysis procedures (Mateescu et al., 2017; Couch et al., 2021). Blood is the initial source of EVs-based biomarkers and is commonly employed in clinical diagnosis (Boukouris and Mathivanan, 2015). It has been established that fresh plasma and serum contain intact exosomes (Muller et al., 2014). A single freeze-thaw cycle with a shorter storage time does not change the size or concentration of EVs. In contrast, repeated freeze/thaw cycles had been shown to cause an increase in protein aggregation (Muller et al., 2014). Additional research has demonstrated that exosomes held in plasma at 80 or 20°C are more stable, providing a better recovery after 90 days than exosomes stored at 4°C (Kalra et al., 2013).

Researchers routinely debate the “optimal” separation approach. While each approach obviously has advantages, the project's objectives influence the decision to adopt one over another. The selection of an isolation method is influenced by several characteristics, such as sensitivity, specificity, sample, cost, and workforce constraints. The most widely used EV isolation and purification procedures, such as differential centrifugation and ultracentrifugation, are time-consuming, take days, and frequently contaminate samples with cellular fragments (Xu et al., 2016). To overcome these issues, recently developed microfluidic devices or platforms can act as an alternative and have been shown to significantly improve the sensitivity and accuracy of EV isolation (Iliescu et al., 2019). These methods allow for faster and higher-throughput isolation of brain-derived EVs. When coupled with advanced techniques, such as enzyme-linked immunosorbent assay (ELISA), next-generation sequencing, and biomarker discovery, these technologies may enable the rapid assessment of multiple biomarkers from extremely small amounts of sample material.

The common identification and quantification techniques for EVs include Electron microscopy and Flowcytometry for morphological characterization and the Western immunoblot technique for identification of the markers of membrane protein (Porro et al., 2015). Nonetheless, methodological consideration and variability in standardization techniques remain a source of inconsistencies in EV recovery (Doyle and Wang, 2019). Diversity within EV populations is one of the most intriguing avenues for discovering TBI biomarkers. Recent articles (Zabeo et al., 2017; Tkach et al., 2018) have explored the limits of previously accepted ideas of an “exosome” as well as the significance of identifying EV subtypes. Different separation strategies can remove a fraction of exosomes containing critical diagnostic information, whether they are tiny or big. However, the isolation of certain exosomal groups is still being explored, and categorization is being defined constantly on a regular basis (Couch et al., 2021).

## Conclusions and future perspectives

Over the past two decades, EVs have transformed from their original categorization as “Garbage bins” of cells into the emergence of promising biomarkers and innovative therapeutic agents in several biological processes. They have also been linked to several NDs' pathogenesis, development, and progression. The emerging research and evidence underscore the growing interest in EVs, implying the possibility that they could produce transformational methods for diagnosis, characterization, and therapy in NDs.

TBI is a complex and heterogeneous injury with varied clinical manifestations and recovery patterns. Neurodegeneration and neuroinflammation may play important roles in the pathology of TBI and other neurological illnesses. Mechanisms involving EVs in the CNS may shed light on the potential involvement of EVs in TBI pathogenesis and progression, which is currently unknown. Reliable TBI biomarkers might improve the diagnosis and management of TBI patients. Identifying individuals at high-risk of neurodegeneration post-TBI is crucial to initiating the therapeutic interventions prior to the onset of irreversible pathological manifestations. EVs as biomarkers have shown considerable promise in the realm of TBI diagnoses.

Additionally, EVs may be utilized to support personalized medicine methods for TBI therapy by performing complementary diagnostics for medications undergoing clinical trials. Biomarkers are required to identify which disease processes contribute the most to a patient's condition to evaluate which treatment is most likely to be beneficial for the patient. Thus, EV-based biomarkers can play an important role for developing personalized medicine to treat patients with TBI.

An increasing body of research indicates that EVs obtained from peripheral blood might be used for the diagnosis of TBI. However, EV research is still at an early stage, as there are methodological constraints with respect to optimal standardization methods, nomenclature, and methodological characterization. Regarding “analytical performance,” critical technological issues will include the development of validated methodologies capable of isolating, classifying, and evaluating EV subtypes accurately and efficiently. Before any more steps can be taken toward clinical use, the results must be reliable, be able to be repeated in different clinical settings, and have higher sensitivity and specificity. In this case, it would be very important to find out if specific exosomes and MVs allow phenotypic characterization of NDs. This could bring new life to the current classification and lead to a novel risk stratification system based on molecular phenotyping and biological and pathophysiological implications. Moreover, larger clinical studies will be required to evaluate and define the clinical utility of particular EV populations within specific contexts and identify its additional value for developing newer methodological approaches to integrate clinical and molecular information to improve outcomes.

Finally, we still need to explore and define the clinical utility of specific EVs subsets within a specific context of use and assess its value beyond current clinical practice while developing new methodological approaches for their integration and combination with other types of clinical and molecular data.

Based on these and other unanswered problems in the field of EVs, we anticipate that the advancement in research will lead to the development of novel diagnostic and treatment approaches for TBI. The current knowledge of exosome composition, biosynthesis, function, and their potential as diagnostic and therapeutic candidates continues to expand, providing new insights into normal physiology and disease processes in neurodegenerative diseases.

## Author contributions

NK, MA, KB, and AE: conceptualization. NK, MA, KB, SR, AE, and HA: methodology and writing—original draft preparation. NK, MA, KB, HA, and AE: data curation. NK, AE, MA, KB, and SR: writing—review and editing. AE, HA, and SR: supervision. All authors have read and agreed to the published version of the manuscript.

## Conflict of interest

The authors declare that the research was conducted in the absence of any commercial or financial relationships that could be construed as a potential conflict of interest.

## Publisher's note

All claims expressed in this article are solely those of the authors and do not necessarily represent those of their affiliated organizations, or those of the publisher, the editors and the reviewers. Any product that may be evaluated in this article, or claim that may be made by its manufacturer, is not guaranteed or endorsed by the publisher.
